# Reconceptualizing audience presence in music performance anxiety: a multilevel framework of external conditions and internal representation

**DOI:** 10.3389/fpsyg.2026.1851626

**Published:** 2026-09-21

**Authors:** Chun Lee

**Affiliations:** Independent Researcher, Hong Kong, Hong Kong SAR, China

**Keywords:** anxiety, audience identity, music performance anxiety, perception, psychological presence

## Abstract

This paper develops a multilevel framework to explain how audience presence shapes music performance anxiety (MPA). It distinguishes between external audience conditions, including Physical Attendance (PA) and Alternative Attendance (AA), and internally generated audience representations, termed Imaginative Attendance (IA). The framework also differentiates audience-related inputs in terms of Externally Assigned Identity (EAI) and Perceived Evaluative Identity (PEI), which are integrated through Perceived Social Evaluation (PSE), a contextual appraisal process. PSE shapes cognitive, emotional, and physiological responses, while metacognitive regulation influences whether these responses contribute to threat-focused processing and social inhibition or task-focused processing and social facilitation.

## 1 Introduction

Music performance anxiety (MPA) remains a substantial concern in music education. A systematic review of undergraduate musicians reported highly variable prevalence estimates, ranging from 16% to 83.1%, and identified interacting cognitive, physiological, behavioural, and contextual predictors (Barros et al., 2022). Recent qualitative and quantitative evidence further indicates that MPA is shaped by contextual predictors such as audience type, auditions, competitive settings, teaching practices, and institutional culture (Barros et al., 2024; Barros et al., 2026). Among these contextual influences, audience-related evaluation is particularly salient because music performance frequently occurs in situations in which performers know that their actions may be observed and judged by others. Audience-related evaluation is therefore an important situational contributor to MPA, although it is neither its sole cause nor uniformly detrimental to performance. Evaluative situations have been associated with heightened physiological activation, attentional disruption, and variability in performance quality (Wan and Huon, 2005; Morin, 2005; Kenny, 2011; Papageorgi et al., 2013). These findings suggest that MPA cannot be understood solely as an individual response detached from the social and evaluative environment in which performance takes place.

Traditional explanations of audience effects have largely been grounded in social-evaluative accounts. Zajonc’s (1965) drive theory proposes that the presence of others increases arousal and strengthens dominant responses, facilitating well-learned actions while impairing difficult ones (Mullen et al., 1997; van Meurs et al., 2024). Evaluation apprehension theory refined this account by arguing that audience effects depend not merely on physical presence, but on the belief that observers can evaluate the individual (Cottrell et al., 1968; Cottrell, 1972; Loomis et al., 1999). Choking-under-pressure accounts further describe how evaluative demands may redirect attention towards errors, consequences, and the conscious control of normally automatic processes (Baumeister, 1984; Westenberg et al., 2009). Together, these perspectives establish that audience effects depend on task demands and performers’ cognitive appraisals rather than on physical presence alone. However, they do not fully explain how different audience conditions acquire subjective, context-specific evaluative meaning for performers.

Established models of MPA provide an important etiological and temporal foundation for the present framework. Barlow’s (2000) emotion-based account proposes that anxiety develops through the interaction of generalized biological vulnerability, generalized psychological vulnerability, and specific learning experiences. Kenny’s (2011) biopsychosocial account applies this perspective to MPA, emphasizing how dispositional susceptibility, developmental and evaluative experiences, and performance-specific conditioning may contribute to anxious apprehension in high-investment performance situations. Papageorgi et al. (2007), in turn, conceptualize MPA as a dynamic process unfolding across pre-performance, performance, and post-performance phases, shaped by performer susceptibility, task efficacy, and the performance environment, and capable of producing either adaptive or maladaptive outcomes. These models explain why performers differ in their vulnerability to MPA and how anxiety develops over time. However, they do not fully differentiate how physical, technologically mediated, or internally represented audiences acquire subjective evaluative meaning. The present framework addresses this more specific explanatory problem.

Recent intervention research further underscores the need for more theoretically specific accounts of MPA. An updated systematic review and meta-analysis found that interventions for performance anxiety in musicians generally produced greater symptom reduction than control conditions, but substantial heterogeneity prevented clear identification of the active mechanisms responsible for improvement (Nicholl and Abbott, 2026). Similarly, a recent systematic review identified potential benefits across cognitive-behavioural therapy, acceptance and commitment therapy, mindfulness, yoga, and other psychological and physical coping approaches, while highlighting small samples and methodological limitations across the evidence base (Bakhtiari et al., 2025). These findings support the value of multifaceted intervention but also indicate a need to specify the contextual, appraisal, and regulatory mechanisms through which particular strategies may become effective. The present framework contributes to this objective by isolating audience-related processes rather than proposing a comprehensive treatment model for MPA.

The need for a more context-specific account has become more pressing as musical performance has expanded beyond face-to-face settings. The COVID-19 pandemic accelerated the adoption of digitally mediated forms of music learning, assessment, and performance, several of which have remained part of post-pandemic practice (Ritchie and Sharpe, 2021; Cheng et al., 2026). Performances and assessments now take place through livestreams, recordings, online auditions, remote examinations, and virtual environments. Recent evidence that virtual performance settings can elicit substantial MPA further highlights the importance of examining how altered audience configurations shape performers’ evaluative experiences (Ryan et al., 2025). Research on presence indicates that remote or represented others may be experienced as psychologically real and socially salient (Lombard and Ditton, 1997; Riva et al., 2003). In such contexts, technological cues may acquire evaluative significance by signalling access to actual, anticipated, or simulated audiences, even when no audience is physically co-present (Williamon et al., 2014). These developments support the development of a framework explaining how different forms of audience presence acquire evaluative meaning. Such a framework can provide a basis for empirical investigation and potentially inform future training, assessment, and intervention strategies across contemporary performance contexts.

The central problem is not that existing theories confine evaluation to physical audiences. Rather, MPA research has often compared live, recorded, and no-audience conditions without sufficiently specifying how these conditions acquire subjective evaluative meaning (LeBlanc et al., 1997; Yoshie et al., 2009; Williamon et al., 2014; Aufegger and Wasley, 2020). Objective format alone does not determine whether a performer feels evaluated. The same camera, for example, may be psychologically neutral during private review but highly evaluative during an examination, while the evaluative significance of a physically present audience may vary across performers and contexts. Audience effects depend on who performers understand the audience to be and what consequences they associate with its judgment.

**Figure 1:**
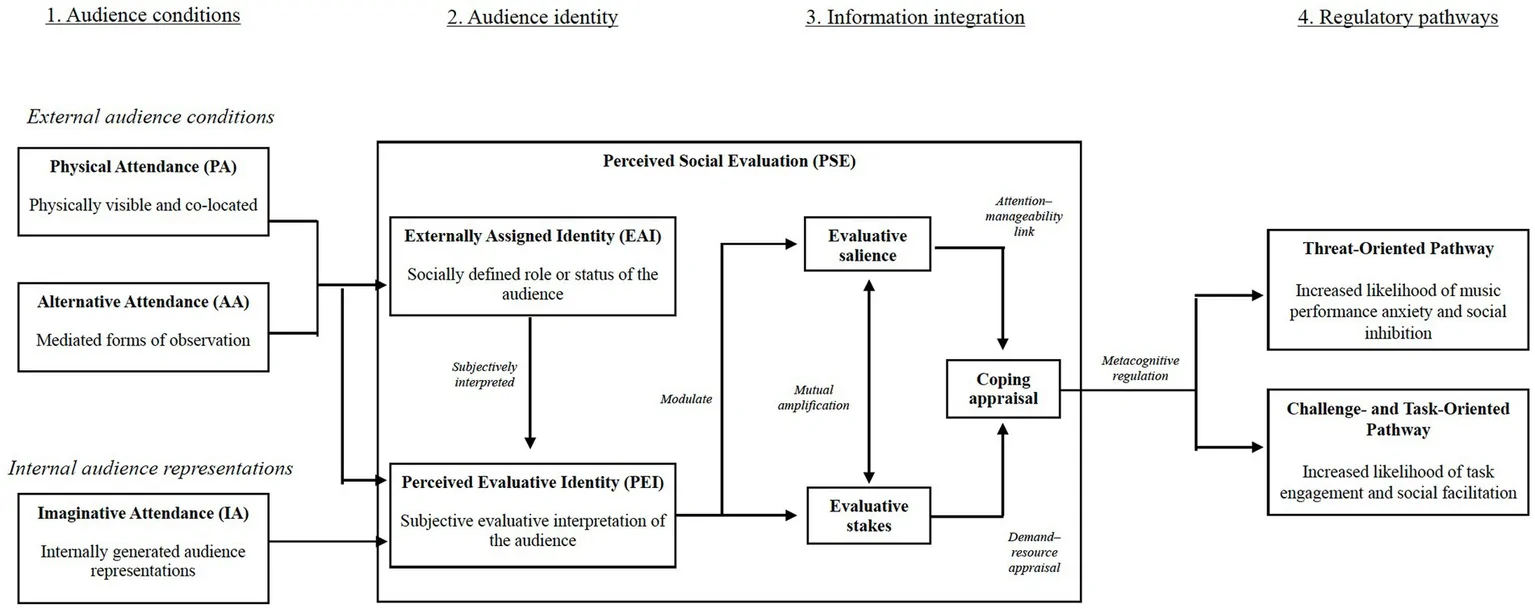
Proposed multilevel framework linking audience presence to music performance responses. Physical attendance (PA), alternative attendance (AA), and imaginative attendance (IA) shape externally assigned identity (EAI) and perceived evaluative identity (PEI), which inform perceived social evaluation (PSE). Through metacognitive regulation, these appraisal processes increase the likelihood of either threat-oriented outcomes or challenge- and task-oriented outcomes.

The absence of an immediate external audience presents a related problem. Cognitive models suggest that evaluative observers can be internally simulated through self-evaluative processes, including self-focused attention, anticipated judgment, and self-evaluation (Wells et al., 1995; Rapee and Heimberg, 1997). Self-regulation frameworks propose that individuals monitor behaviour against internalized standards (Carver and Scheier, 2012), while theories of self-presentation and imagined audiences indicate that people can represent how they appear from the perspective of socially significant others (Elkind, 1967; Leary, 1992; Decety and Grèzes, 2006). Such representations are associated with attentional bias and anxiety under evaluative conditions (Eysenck et al., 2007; Moriya and Sugiura, 2012), including situations without an immediate observer (Mor and Winquist, 2002). A no-audience condition therefore cannot automatically be treated as psychologically neutral.

To address this gap, the general objective of this paper is to develop a multilevel framework that explains how external audience conditions and internally represented audiences shape performers’ arousal and performance-related responses through contextual appraisal and metacognitive regulation. Situated within these established MPA models, the present framework draws additionally on Social Cognitive Theory (Bandura, 1986) and Cognitive Appraisal Theory (Lazarus and Folkman, 1984; Lazarus, 1991) to specify how audience-related experiences are learned, internally represented, appraised, and regulated. The framework positions performers as active interpreters of audience-related information rather than passive recipients of environmental stimuli. Rather than treating the audience as a uniform external factor, it distinguishes among different forms of audience presence and explains how audience-related inputs acquire context-specific evaluative meaning.

To achieve this general objective, the paper pursues three specific objectives: (1) to differentiate Physical Attendance (PA), Alternative Attendance (AA), and Imaginative Attendance (IA), and to explain how these dimensions interact and are recursively updated across performance experiences; (2) to specify how audience identity is construed through Externally Assigned Identity (EAI) and Perceived Evaluative Identity (PEI), and how these forms of identity are integrated through Perceived Social Evaluation (PSE), including evaluative salience, evaluative stakes, and coping appraisal; and (3) to explain how metacognitive regulation influences whether PSE-related cognitive, emotional, and physiological responses contribute to threat-focused processing and social inhibition or to task-focused processing and social facilitation. These objectives provide a roadmap for the theoretical development that follows and are operationalized through the seven propositions presented in Section 2.4.

## 2 Theoretical framework

### 2.1 Audience presence dimensions

The present framework conceptualizes audience presence as a multidimensional construct involving both external audience conditions and internally generated psychological representations. The perception of being observed or evaluated may arise through different combinations of spatial proximity, temporal immediacy, perceptual access, and psychological salience. Accordingly, audience presence is divided into Physical Attendance (PA), Alternative Attendance (AA), and Imaginative Attendance (IA). PA and AA refer to external audience conditions, whereas IA refers to an internally generated representation of an observing or evaluating audience. These dimensions are not mutually exclusive, as imagined audiences may coexist with either physical or mediated attendance.

#### 2.1.1 Physical Attendance (PA)

Physical Attendance (PA) refers to performance situations in which performers and audience members occupy the same physical environment at the same time. Its defining characteristics are spatial co-presence and temporal simultaneity. PA includes conventional live-performance contexts such as concerts, recitals, auditions, competitions, and classroom performances in which performers and observers share an immediate physical setting.

Physical co-presence does not necessarily involve direct interaction, complete audience visibility, or active participation. Depending on venue design, stage lighting, seating distance, and established performance conventions, performers may have limited visual or interpersonal access to audience members. Nevertheless, the audience may remain perceptible through environmental and behavioural cues, including gaze, posture, movement, silence, breathing, and applause (Kawase, 2014; Bradby, 2016). These cues distinguish PA from forms of audience presence in which observers are spatially or temporally separated from the performer.

A key feature of PA is the possibility of immediate and reciprocal influence. Because performers and audiences share the same environment, audience responses may occur during or directly after the performance. These responses can be explicit, such as applause, facial expressions, or visible gestures, or more subtle, such as changes in attention, bodily movement, and collective silence. The performer’s behaviour may likewise affect audience attention and responsiveness, producing a dynamic form of interpersonal engagement (Gallagher, 2001; De Jaegher and Di Paolo, 2007).

Research suggests that physically present audiences can influence performers’ psychological responses, physiological arousal, attention, and behaviour (Bereczkei et al., 2010; Epting et al., 2011; Spohn et al., 2022). However, MPA is a multidimensional phenomenon involving interacting psychological, cognitive, physiological, behavioural, and contextual factors and cannot be attributed to audience presence alone (Barros et al., 2022). Qualitative evidence from higher music education further indicates that performers’ anxiety varies according to audience type, performance venue, solo or group format, and the presence of competitive or jury-based evaluation. Performers may also interpret their arousal as either facilitative or debilitating (Barros et al., 2024). Consistent with this evidence, evaluation apprehension theory proposes that audience effects are especially likely when observers are perceived as capable of evaluating the performer, rather than arising from physical presence alone (Cottrell et al., 1968). These accounts support a clear distinction between PA, perceived evaluation, and performance anxiety. PA describes an externally observable audience condition, while the evaluative significance attributed to that condition may vary across performers and contexts.

Naturalistic PA settings may also be difficult to standardize experimentally because audience size, composition, familiarity, visibility, and behaviour can differ across performances. Although standardized audiences may improve experimental control, they may reduce the spontaneity and ecological validity associated with live performer–audience interaction.

#### 2.1.2 Alternative Attendance (AA)

Alternative Attendance (AA) refers to performance situations in which audiences are not physically co-present with the performer but access the performance through technological mediation. AA includes formats such as livestreaming, video conferencing, recorded examinations, online auditions, and virtual audience simulations. Its defining feature is the presence of an external technological medium that connects the performance to an actual, anticipated, or simulated audience.

The psychological significance of AA can be understood through the broader concept of presence and, more specifically, social presence. Lee (2004) conceptualizes presence as a psychological state in which mediated or virtual entities are experienced as if they were actual entities and distinguishes among physical, social, and self-presence. For the present framework, this account locates the psychological effects of mediation in the performer’s experience rather than in the properties of the technological medium alone. Social presence refers more specifically to the extent to which mediated others are experienced as psychologically real and socially salient (Short et al., 1976; Biocca and Harms, 2002). Although technological mediation may reduce direct sensory and interpersonal access, mediated others can still elicit meaningful cognitive, emotional, and behavioural responses (Biocca et al., 2003; Reader and Holmes, 2016). In AA contexts, cameras, recording indicators, viewer counts, comments, avatars, and platform interfaces can function as audience-related cues by signalling current or future access to the performance. The availability of these cues alone, however, does not guarantee social presence or evaluative significance. Their meaning depends on what performers believe they indicate about observation, audience access, evaluative authority, and future consequences (Pavis, 2003).

AA varies along three principal dimensions: temporal structure, audience status, and reciprocity. Temporal structure concerns whether audience access occurs synchronously or asynchronously. In synchronous contexts, such as interactive livestreams or live remote auditions, audiences observe the performance as it unfolds and may provide immediate responses. In asynchronous contexts, such as recorded examinations or pre-recorded auditions, audiences view or evaluate the performance later. Although evaluators are not co-present in asynchronous contexts, anticipated evaluation can still influence performance. Experimental evidence indicates that anticipated evaluation affects responses independently of the physical presence of an audience (Paulus and Murdoch, 1971). Consistent with this account, university pianists reported significantly lower MPA during recorded performances distributed to an absent online audience than during live performances, although they still experienced some mental symptoms of anxiety (Conklin, 2011). Similarly, research on asynchronous video interviews shows that recorded responses can involve interview anxiety and impression-management concerns (Rizi and Roulin, 2024).

Audience status distinguishes actual remote audiences from simulated audiences. Actual remote audiences consist of identifiable or unidentified people who access the performance through mediated channels, either synchronously or asynchronously. Simulated audiences are technologically generated representations, such as virtual spectators, juries, or avatars. Virtual environments may generate spatial presence, referring to the experience of being located within a mediated setting, and social presence, referring to the experience of being with socially meaningful others (Slater and Wilbur, 1997; Lee, 2004). Simulated audiences can elicit meaningful emotional, attentional, physiological, and behavioural responses, although the strength of these responses depends on the characteristics of both the simulation and the performance context (Stevens and Kincaid, 2015; Murray et al., 2016).

Recent music-specific research provides further support for treating simulated audience conditions as variable rather than uniform. In an immersive virtual auditorium, Gómez-Sirvent et al. (2024) found that ambient lighting and distance from the virtual audience altered musicians’ perceived exposure, appraisal of the environment, and gaze behaviour. These findings suggest that the evaluative salience of a simulated audience depends not only on whether avatars are present, but also on how visible, proximal, and attentionally prominent they are. Bellinger et al. (2023) have additionally developed a randomized controlled trial protocol comparing virtual-reality exposure with progressive muscle relaxation for musicians with performance anxiety. The protocol incorporates subjective anxiety measures together with heart-rate variability, blood pressure, cortisol, and other physiological indicators. Because this publication reports a study protocol rather than completed trial findings, it demonstrates the methodological and clinical development of VR-based MPA research but does not yet establish the efficacy of the intervention. Simulated audiences should therefore be understood as controlled sources of audience-related information that may reproduce some, but not necessarily all, of the evaluative properties of physical attendance.

Evidence comparing live and screen-mediated performance also indicates that the two modes should not be treated as psychologically interchangeable. Gabriel et al. (2025) found that viewing a live performance and viewing the same performance on a screen under otherwise comparable conditions produced different subjective and physiological patterns, with the live condition generally eliciting stronger audience engagement. Because the physiological measures were obtained from audience members rather than performers, these findings do not directly demonstrate differences in performers’ MPA. They nevertheless suggest that physical co-presence can alter audience engagement and, consequently, the reciprocal social cues potentially available to performers. This provides a further reason to distinguish PA from AA rather than treating technological mediation as a functionally equivalent substitute for physical attendance.

Reciprocity refers to the extent to which audience responses are contingent on the unfolding performance. Interactive formats may provide immediate and performance-contingent feedback through comments, visible reactions, gestures, or adaptive virtual behaviour. Such responsive cues may strengthen social presence when they are experienced as appropriately connected to the performer’s actions (Oh et al., 2018). Non-interactive formats, including recordings and pre-programmed simulations, do not allow performers to verify or update audience responses in real time. The availability and timing of interpersonal information depend partly on media characteristics such as visibility, audibility, contemporality, and transmission velocity (Clark and Brennan, 1991; Dennis et al., 2008). Technological mediation can filter, delay, or restrict such information, with the resulting degree of ambiguity varying across settings.

AA may reduce some pressures associated with one-time physical performance, particularly when performers can repeat takes or select a preferred recording. At the same time, the possibility of delayed, repeated, or widely distributed observation may increase the perceived permanence and reach of evaluation (Bay-Cheng et al., 2010). AA is therefore defined not by a single technological format, but by the interaction of temporal structure, audience status, reciprocity, and the audience-related information made available through mediation.

#### 2.1.3 Imaginative Attendance (IA)

Imaginative Attendance (IA) refers to the internally generated or reactivated mental representation of an audience. Unlike PA and AA, IA does not require either an audience or an audience-related medium to be present in the immediate environment. It occurs when performers mentally represent specific individuals or a generalized audience whose judgment is anticipated.

IA is related to, but distinct from, Elkind’s (1967) concept of the imaginary audience and Cooley’s (1922) looking-glass self. Elkind’s imaginary audience was originally developed to explain perceived observation associated with adolescent egocentrism, whereas IA is proposed here as a performance-related process that may occur across developmental stages. Cooley’s looking-glass self-concerns the broader formation of self-concept through imagined social judgment, whereas IA has a narrower focus on the temporary representation of an audience within a specific performance or rehearsal context.

IA is informed by previous and anticipated social experiences. Its characteristics may vary according to the vividness of imagery, the frequency and emotional intensity of previous evaluative encounters, and individual sensitivity to social judgment (Cocquyt and Palombo, 2023). IA may arise spontaneously or it may be deliberately constructed.

IA may occur independently or coexist with PA and AA. Moreover, these audience sources may involve different audience identities, a point that will be further discussed in Section 2.3.1 *Audience identity as an interpretive framework*. For this reason, IA cannot be inferred solely from the objective audience condition. Its consequences may be adaptive or maladaptive: a deliberately constructed supportive or realistic audience representation may assist preparation, concentration, and psychological rehearsal (Sheikh and Korn, 1994; Watkins, 2008; Sweller et al., 2011), whereas representations of highly critical or threatening evaluators may increase performance anxiety (Watkins, 2008; Ng and Abbott, 2014). IA thus challenges the assumption that no-audience conditions are psychologically neutral, while remaining conceptually distinct from the appraisal and regulatory processes discussed later in the framework.

The three dimensions of audience presence are summarized in Table 1. Although they differ in source, they interact dynamically.

**Table 1:** Comparative summary table.

Dimension	Spatial relationship	Source of evaluation	Mode of presence	Temporal characteristics	Mediation
Physical Attendance (PA)	Co-located	External observers	Physical co-presence	Immediate and synchronous	None
Alternative Attendance (AA)	Spatially separated	Technologically mediated external observers	Mediated presence	Synchronous or asynchronous	Technological
Imaginative Attendance (IA)	No external co-presence	Internally represented observers	Mental representation	Variable	Internal cognitive

### 2.2 The interaction among PA, AA, and IA

#### 2.2.1 Driving mechanisms across audience presence dimensions

The interaction among PA, AA, and IA operates through both bottom-up and top-down processes. External audience conditions provide social, environmental, and technological cues that may activate or update internal audience representations. At the same time, previously established representations may shape performers’ attention to and interpretation of current audience-related information. Three mechanisms are proposed: mediated activation, internalization, and top-down reinterpretation. Together, these mechanisms form a recursive updating cycle across performance experiences.

##### 2.2.1.1 Mediated activation of IA

Mediated activation occurs when cues associated with AA evoke a mental representation of an absent, remote, future, or simulated audience. According to evaluation apprehension theory, arousal is associated not simply with the presence of others, but with the expectation that one’s behaviour may be evaluated (Cottrell, 1972). A camera, recording indicator, online submission platform, or livestream interface can function as a proxy for an audience by signalling that the performance is accessible to evaluators. During a recorded examination, for example, the examiners are absent at the time of performance, but the performer may mentally represent them while facing the camera because the recording device signifies future observation and judgment. Social anxiety models similarly suggest that individuals can construct an internal observer perspective that remains psychologically active in the absence of physically present observers (Rapee and Heimberg, 1997). AA cues may therefore activate IA and generate evaluative concern even when no audience is immediately visible.

The strength of mediated activation is likely to depend on the clarity and salience of the connection between the technological cue and its anticipated audience. A camera used for private technical review may evoke a different representation from one used for a formal examination or public broadcast. The device itself does not possess evaluative authority; rather, it signals access by an audience to whom the performer attributes evaluative significance.

##### 2.2.1.2 Internalization of audience experience

Internalization refers to the process through which experiences with PA or AA are encoded into relatively enduring audience representations that can later be reactivated as IA. From a social cognitive perspective, social experiences may be symbolically represented and retained as cognitive models that guide subsequent perception and behaviour (Bandura, 1986). Repeated encounters with audiences can shape how performers subsequently represent observation, judgment, support, and threat.

This internalization process, however, is not inherently adaptive. Exposure and inhibitory-learning accounts suggest that repeated engagement with evaluative situations alters existing cognitive models by providing new data regarding threat, personal coping ability, and situational outcomes (Foa and Kozak, 1986; Craske et al., 2014). Consequently, what a performer learns from these encounters dictates the nature of the representation. While repeated supportive or manageable experiences may foster predictable, less threatening representations, encounters characterized by criticism, embarrassment, or perceived failure tend to strengthen threat-oriented models, thereby maintaining or intensifying performance anxiety (Candia et al., 2023).

Preparation methods such as dress rehearsals, simulated performances, and recorded practice may provide structured opportunities for representational updating by exposing performers to environmental and evaluative features such as venue acoustics, stage layout, audience proximity, and recording procedures. Information learned about these features may be incorporated into stored audience representations and later reactivated as IA during preparation or performance. Internalization captures the mechanism through which past external audience encounters continue to shape performers’ appraisals and responses after the original situation has ended.

##### 2.2.1.3 Top-down reinterpretation of external audience cues

Top-down reinterpretation occurs when activated IA shapes how performers attend to and interpret current PA or AA cues. Mental imagery may evoke emotional and physiological responses resembling those associated with actual events (Lang, 1979; Holmes and Mathews, 2010). When audience representations are vivid or emotionally salient, they may direct attention towards information that is consistent with the performer’s existing expectations. For example, a performer who has internalized a highly critical audience may interpret silence, limited facial expression, or delayed online feedback as evidence of disapproval. Another performer with a more supportive audience representation may interpret the same cues as concentration, neutrality, or respectful attention. IA can shape the meaning assigned to ambiguous audience behaviour rather than merely reproducing previous experience.

Although Clark and Wells (1995) cognitive model concerns social phobia and observer-perspective representations of the self rather than audience representations, it identifies a relevant cognitive mechanism. Perceived social-evaluative threat may shift attention towards internally generated information about how the self appears to others. Observer-perspective self-images and anticipated judgments may be treated as socially informative even when external feedback is limited or ambiguous. Applied to music performance, this mechanism helps explain how IA can guide performers’ attention and influence their interpretation of subsequent PA or AA cues.

This process can operate in both PA and AA contexts. During a live concert, an internalized audience representation may shape how the performer interprets the behaviour of physically present listeners. During a livestream or recorded examination, the same representation may influence responses to viewer counts, interface indicators, cameras, or delayed feedback. Broader imagery research indicates that mental representations can influence attentional focus and emotional preparation (Cumming and Williams, 2012; Moran and O'Shea, 2020). IA is defined more narrowly as audience-related imagery rather than mental practice in general, with its proposed role centred on the interpretation of external audience cues and preparation for anticipated evaluation.

#### 2.2.2 Recursive updating cycle

The three mechanisms form a recursive cycle across time. Experiences involving PA or AA may activate and modify IA, while IA influences how later physical or mediated audience conditions are interpreted. These interpretations then become part of subsequent experience and may further update the performer’s internal audience representations.

This cycle is consistent with reconstructive accounts of memory, according to which past experiences are not stored and retrieved as exact records but are reconstructed through existing beliefs, expectations, and schemas (Bartlett, 1932; Beck, 1976). Audience encounters may therefore be remembered in ways that shape subsequent expectations about future audiences. When such encounters are interpreted as threatening or rejecting, they may strengthen threat-oriented expectations, which can subsequently increase attention to ambiguous signs of rejection and reinforce existing audience representations. Conversely, when encounters contradict previous expectations or are interpreted more positively, they may gradually contribute to more differentiated, flexible, or less threatening representations of future audiences. This recursive account suggests that psychological effects emerge through sustained interactions across diverse performance contexts, as audience-related experiences may either reinforce or revise preexisting audience representations.

### 2.3 Perceived Social Evaluation (PSE) as an integrative appraisal system

The present framework introduces Perceived Social Evaluation (PSE) to explain how different forms of audience presence and audience representation are transformed into a context-specific experience of being evaluated. PSE is defined as the performer’s subjective appraisal that the self or the performance is being, or may be, evaluated by one or more audience sources, including PA, AA, and IA. This definition refers to perceived evaluation by a socially represented other, rather than self-assessment based solely on private personal standards.

Drawing on Cognitive Appraisal Theory (Lazarus, 1991; Lazarus and Folkman, 1984) and Social Cognitive Theory (Bandura, 1986), the PSE construct positions performers as active interpreters of audience-related situations. Their responses arise from appraisals of situational meaning and reciprocal interactions among environmental cues, personal beliefs, and behaviour. PSE is related to, but distinct from, evaluation apprehension, self-focused attention, and social anxiety. Clark and Wells’s (1995) cognitive model is relevant because it identifies self-focused attention, observer-perspective self-representations, and anticipated social judgment as mechanisms through which evaluative threat becomes psychologically salient (see also Mor and Winquist, 2002; Rapee and Heimberg, 1997). However, the Clark and Wells model primarily concerns processes that maintain negative social-evaluative anxiety, whereas PSE is proposed as a broader, context-sensitive appraisal construct. PSE refers to the performer’s appraisal that an audience source is evaluatively relevant to the self or performance, regardless of whether the anticipated evaluation is negative, positive, or uncertain. Self-focused attention and observer-perspective imagery may contribute to the formation or intensification of PSE, but neither is equivalent to the construct itself.

Within this framework, audience presence and audience representations are not assumed to produce uniform psychological responses. They provide external cues or internal representations whose effects depend on how they are appraised by the performer. PSE is proposed as the proximal appraisal process linking these audience-related inputs to subsequent cognitive, emotional, physiological, and behavioural responses.

The structure of PSE consists of three distinct but interrelated dimensions: evaluative salience, evaluative stakes, and coping appraisal.

#### 2.3.1 Evaluative salience

Evaluative salience refers to the degree to which possible audience evaluation is experienced as noticeable, relevant, and central to the performer’s immediate goals. It reflects whether evaluation occupies a prominent position within the performer’s awareness during a particular performance situation. Salience is influenced by the availability of audience-related cues and by the performer’s construction of audience identity: an audience may be physically present without becoming highly salient, whereas an absent evaluator represented through a camera, recording platform, or imagined social figure may become the performer’s primary focus. For example, an explicitly identified examination panel may acquire high salience when its members are perceived as authoritative or professionally consequential. High evaluative salience increases the likelihood that attention will be directed towards possible observation and judgment, although salience alone does not determine whether the experience will be threatening or facilitative (Lazarus, 1991; Oliver and Brough, 2002).

#### 2.3.2 Evaluative stakes

Evaluative stakes refer to the perceived importance and anticipated consequences of audience judgment. This dimension concerns what the performer believes may be gained, lost, confirmed, or threatened through evaluation. Relevant consequences may include examination results, professional opportunities, social approval, artistic credibility, or the fulfilment of personal and interpersonal expectations. Evaluative stakes are not determined solely by the objective performance context. The same recital may be experienced as relatively low-stakes by one performer but as highly consequential by another. Stakes depend partly on credibility of the audience. They may also be amplified in mediated contexts when a performance can be stored, assessed repeatedly, or distributed to audiences beyond the immediate situation.

Evaluative salience and evaluative stakes are related but conceptually distinct: a performer may be acutely aware of possible evaluation while regarding its consequences as relatively minor (e.g., a busker noticing the reactions of passing pedestrians), whereas an evaluator may be only minimally perceptible while their anticipated judgment carries substantial consequences (e.g., an audition panel positioned behind a screen).

#### 2.3.3 Coping appraisal

Coping appraisal refers to the performer’s judgment of whether available resources are sufficient to meet the perceived evaluative demands of a performance situation. Consistent with secondary appraisal theory, such resources may include technical preparation, prior performance experience, self-efficacy, familiarity with the setting, and access to social or practical support (Lazarus and Folkman, 1984). This appraisal concerns the perceived adequacy of these resources, rather than the regulatory processes through which they are later deployed. Self-efficacy is especially relevant because beliefs about personal capability shape whether evaluative demands are interpreted as manageable (Bandura, 1986). When performers perceive their resources as sufficient, even high-stakes evaluation may be appraised as a manageable challenge that supports motivation and engagement; when perceived demands exceed resources, the same situation is more likely to be appraised as threatening and to increase evaluation apprehension.

The three dimensions of PSE interact and may change throughout preparation and performance. Audience responses can alter how salient, consequential, or manageable evaluation is perceived to be. PSE is conceptualized as a context-sensitive appraisal state rather than a fixed personal trait. This structure offers a possible explanation for why objectively similar audience conditions may produce different responses. Two performers may regard the same audience as equally salient and its judgment as equally consequential yet differ in their perceived capacity to cope with that judgment. By distinguishing these dimensions, PSE specifies how audience-related conditions can acquire subjective evaluative meaning without treating audience presence as a uniform or direct cause of MPA.

#### 2.3.4 Audience identity as an interpretive framework

In the framework of PSE, audience identity serves as a central interpretive component. Rather than treating authority, status, expertise, and legitimacy as separate mechanisms, the framework consolidates these characteristics under audience identity because the meaning of audience-related cues depends entirely on how the performer perceives them. Consequently, audience identity modulates the evaluative salience and stakes of the performance because a more authoritative or consequential audience amplifies the perceived importance of their judgment.

##### 2.3.4.1 Dual structure of audience identity

The framework distinguishes between Externally Assigned Identity (EAI) and Perceived Evaluative Identity (PEI).

###### 2.3.4.1.1 Externally Assigned Identity (EAI)

Externally Assigned Identity (EAI) refers to the socially specified or contextually recognisable role attributed to an audience. Examples include an examiner, teacher, peer, critic, competition judge, general listener, or anonymous online viewer. Established primarily by the external performance context, EAI conveys the audience’s expected function and relationship to the performer and may be explicitly designated in PA or AA contexts. The construct captures the socially recognisable category assigned to an audience rather than the immediate psychological meaning attributed to it by the performer.

###### 2.3.4.1.2 Perceived Evaluative Identity (PEI)

Perceived Evaluative Identity refers to the performer’s subjective construction of the audience’s evaluative character and significance. It includes perceptions of the audience’s evaluative intentions. It represents how an externally assigned or internally represented audience is psychologically experienced by the performer. PEI is shaped by prior evaluative experience, expectations, current audience-related cues, and the performer’s interpretation of the performance context. In IA and asynchronous AA, it is formed primarily through anticipation, memory, and internal simulation. In PA and reciprocal forms of AA, PEI may also be revised through immediate audience behaviour.

EAI and PEI should be clearly distinguished. Whereas EAI is often relatively stable, PEI is more context-sensitive and may shift during performance. In reciprocal contexts, social meaning may emerge through ongoing interpersonal engagement rather than detached inference alone, consistent with second-person approaches to social cognition (Gallagher, 2001; De Jaegher and Di Paolo, 2007). During PA and synchronous, interactive forms of AA, performers may revise PEI in response to perceptible audience behaviour while EAI remains unchanged. A judge, for example, retains the same role at the level of EAI but may be construed as supportive, neutral, or critical on the basis of visual and auditory cues. Such cues can alter how performers interpret the audience’s evaluative stance.

##### 2.3.4.2 Integration of multiple audience identities

Performers may encounter or represent several audiences’ identities simultaneously within the three dimensions of audience presence, each associated with different identities. The contribution of each audience source to PSE is proposed to depend on two related principles: relative weighting and evaluative congruence.

##### 2.3.4.2.1 Relative weighting

Relative weighting refers to the degree of influence assigned to each audience identity, where identities with greater influence contribute more strongly to evaluative salience and stakes. This proposal aligns with social impact theory, which suggests that social influence varies based on factors like source strength and immediacy (Latané, 1981). Furthermore, an audience’s orientation influences this dynamic because a supportive orientation can reduce perceived threat or frame high-stakes evaluations as constructive, whereas a critical orientation can increase evaluative salience and stakes to trigger evaluation apprehension. These psychological responses arise through the process of PSE rather than directly from audience identity itself. Nonetheless, this weighting is not static because factors such as audience behaviour, shifting performance contexts, or changes in anticipated consequences can continuously alter the importance attributed to a particular audience source.

###### 2.3.4.2.2 Evaluative congruence

Evaluative congruence refers to the extent to which the PEIs associated with different audience sources indicate similar evaluative orientations. When several highly weighted audience representations are perceived as similarly characteristic, the consistency and certainty of perceived evaluation may increase. Drawing on identity-consistency and self-complexity accounts, the framework proposes that congruent audience representations may reinforce a dominant interpretation, whereas incongruent representations may weaken it or create evaluative uncertainty (Linville, 1985; Burke, 2006; Swann, 2012). Incongruence does not necessarily reduce evaluative pressure. A supportive physical audience may partially offset a critical IA, but conflicting identities of similar weight may also increase uncertainty and attentional conflict. Where one identity carries substantially greater authority or consequences, that identity may dominate PSE despite the presence of other, less influential representations.

Together, Relative weighting and Evaluative congruence allow multiple audience identities to be integrated into an overall evaluative state that may be consistent, conflicted, or uncertain. This integration contributes primarily to evaluative salience and evaluative stakes, while coping appraisal remains dependent on performers’ perceived capacities and available resources.

#### 2.3.5 Metacognitive regulation of PSE: pathways to social facilitation and social inhibition

Audience-related arousal has traditionally been understood as capable of either facilitating or disrupting performance, depending on task mastery and perceived evaluative pressure (Zajonc, 1965; Cottrell, 1972; Kenny, 2009). Within the present framework, PSE is understood as the appraisal process through which audience-related cues and representations are transformed into a context-specific experience of being evaluated.

Metacognitive regulation refers to the processes through which performers monitor, interpret, select, and adjust their reactions to evaluative appraisals during preparation and performance. Whereas PSE concerns the experience of being evaluated, metacognitive regulation concerns how performers manage that experience. Within the present framework, metacognitive regulation is proposed to moderate the relationship between PSE and subsequent cognitive, emotional, physiological, and behavioural responses. Relevant processes may include monitoring and reframing evaluative thoughts, directing attentional focus, interpreting physiological arousal, and adjusting performance strategies in relation to established artistic and task goals (Zimmerman, 2000; Concina, 2019). The perceived success or failure of these regulatory efforts may also shape coping appraisal in subsequent performance situations.

Metacognitive regulation does not independently determine performance outcomes, which remain shaped by broader individual and contextual conditions. Greater monitoring is also not necessarily beneficial. Excessive, rigid, or poorly timed regulation may intensify self-focused attention and interfere with automatic performance processes (Baumeister, 1984; Beilock and Carr, 2001). Effective regulation depends on the flexible deployment of attention and strategies in response to changing performance demands. Within this framework, two probabilistic pathways are proposed: a threat-oriented pathway, in which ineffective or inflexible regulation increases the likelihood of social inhibition, and a challenge- and task-oriented pathway, in which effective regulation supports task engagement and increases the likelihood of social facilitation.

##### 2.3.5.1 Threat-oriented pathway

The threat-oriented pathway is more likely when evaluation is highly salient, the perceived stakes are substantial, and coping appraisal indicates that available resources are insufficient. Under these conditions, ineffective or inflexible metacognitive regulation may allow attention to become dominated by anticipated judgment, bodily symptoms, and possible errors. Objective self-awareness theory suggests that heightened self-focus promotes comparison between the self and perceived standards, particularly when evaluation is prominent (Duval and Silvia, 2002; Phillips and Silvia, 2005). When performers have difficulty regulating this process, discrepancy monitoring may develop into persistent error avoidance, defensive self-monitoring, and concern about negative judgment.

Physiological activation may consequently be interpreted as evidence of distress, declining control, or impending failure. This interpretation may intensify anxiety and increase stimulus-driven attention towards bodily sensations, possible mistakes, and audience reactions. Such processes can reduce attentional control and interfere with automatic motor execution, particularly during complex or highly practised tasks (Baumeister, 1984; Eysenck et al., 2007). Social inhibition in this framework refers specifically to impairment in performance execution under socially evaluative conditions. It is related to, but not equivalent to, the subjective experience of MPA.

##### 2.3.5.2 Challenge- and task-oriented pathway

The challenge- and task-oriented pathway is more likely when performers appraise evaluative demands as important but manageable. Under these conditions, metacognitive regulation helps performers deploy available resources by maintaining goal-directed attention, limiting the disruptive influence of threat-related processing, reinterpreting physiological activation, and adjusting strategies in line with technical and expressive goals (Eysenck et al., 2007). Rather than becoming dominated by error avoidance or anticipated judgment, performers may direct attention toward technical execution, expressive intention, and artistic communication. The audience may then be construed as a legitimate recipient, evaluator, or participant in the performance, rather than solely as a source of threat.

Physiological activation may also be interpreted as manageable or useful. Arousal reappraisal research suggests that viewing activation as supportive of performance can enhance attentional engagement and readiness for action (Jamieson et al., 2010, 2013). In this pathway, arousal may support effort, concentration, expressive intensity, and behavioural preparation rather than signalling loss of control.

This account complements rather than replaces traditional response-dominance explanations of social facilitation. Task familiarity and difficulty remain important, but performance outcomes are also shaped by how performers regulate perceived evaluation and allocate attention during performance. The threat- and task-oriented pathways are not mutually exclusive; performers may shift between them as audience reaction. Metacognitive regulation may help identify these shifts and redirect attention toward task-relevant and expressive goals.

Figure 1 outlines the conceptual framework, illustrating how external and internal audience inputs are integrated through PSE to drive subsequent performance responses under metacognitive regulation.

### 2.4 Theoretical propositions

The proposed framework generates seven theoretical propositions that operationalize the three specific objectives stated in the Introduction. Propositions 1–3 address the differentiation and dynamic interaction of the three audience-presence dimensions; Propositions 4–5 specify the roles of audience identity and PSE; and Propositions 6–7 describe the threat-oriented and task-oriented regulatory pathways. Together, these propositions translate the framework’s central claims into empirically testable relationships among audience-related inputs, appraisal processes, metacognitive regulation, and performance-related responses.

#### 2.4.1 Audience presence and evaluative appraisal

Proposition 1: Differentiated Audience Input. PA, AA, and IA will make distinguishable contributions to performers’ PSE because they provide different forms of audience-related information, respectively through physical co-presence, technological mediation, and internally generated audience representation.

Proposition 2: Imaginative Attendance and Evaluative Salience. When immediate PA and AA are absent or weak, greater vividness, persistence, and evaluative specificity of IA will predict higher evaluative salience. This association will be stronger when the IA is perceived as critical, authoritative, or consequential.

#### 2.4.2 Dynamic interaction over time

Proposition 3: Recursive Updating of Audience Representations. Experiences with PA and AA will modify later IA by strengthening, weakening, or differentiating internal audience representations. In turn, existing IA will shape how performers interpret subsequent physical and mediated audience cues.

#### 2.4.3 Audience identity and PSE

Proposition 4: From EAI to PEI. The effect of an audience’s EAI on evaluative salience and evaluative stakes will be mediated by the performer’s PEI. Specific audiences’ identities will increase perceived evaluation primarily when performers construe them as evaluatively authoritative or consequential.

Proposition 5: Appraisal Mediation of Audience Effects. The effects of PA, AA, and IA on MPA and performance-related responses will be mediated by the three dimensions of PSE: evaluative salience, evaluative stakes, and coping appraisal.

#### 2.4.4 Threat and facilitation pathways

Proposition 6: Threat-Oriented Pathway. When evaluative salience and evaluative stakes are high and coping appraisal is low, performers will be more likely to experience threat-oriented processing, including self-focused attention, error monitoring, negative arousal interpretation, and MPA. These processes will increase the likelihood of performance inhibition.

Proposition 7: Task-Oriented Regulatory Pathway. Flexible, task-focused metacognitive regulation will weaken the relationship between perceived social evaluation and performance inhibition by redirecting attention from anticipated judgment toward task execution, expressive intention, and manageable interpretations of physiological arousal.

## 3 Methodological considerations

To empirically examine the proposed framework, PA, AA, and IA must be operationalized as measurable constructs. This section outlines methodological approaches for experimentally inducing and assessing these dimensions, with the aim of enabling systematic investigation of their individual and interactive effects.

### 3.1 Accounting for background vulnerabilities and temporal processes

Empirical tests of the framework should assess both audience-related processes and the background vulnerabilities identified in established models of MPA. Indicators of trait anxiety or behavioural inhibition, perceived control, performance self-efficacy, perfectionistic concerns, previous negative evaluative experiences, and performance-specific learning history may be examined as antecedents or moderators of IA, PEI, and PSE. Measurements should, where possible, distinguish anticipatory, during-performance, and post-performance responses. This would allow the audience-related mechanisms proposed here to be tested within, rather than independently of, broader developmental and temporal accounts of MPA.

### 3.2 Operationalizing audience-presence conditions

Audience presence is commonly used in experimental music research to induce controlled levels of MPA. In performance contexts, musicians often perform differently from practice, even when the material has been fully mastered. Errors that rarely occur in rehearsal may emerge under evaluative conditions, leaving performers feeling disoriented or unprepared. Simulated anxiety induction enables researchers to systematically examine performers’ responses to evaluative pressure and to investigate factors that enhance resilience in performance settings. Accordingly, audience presence is often implemented as an experimental manipulation to vary perceived levels of social evaluation across conditions.

#### 3.2.1 Inducing physical attendance (PA)

PA is frequently manipulated through audience-related variables to examine how direct observation influences performers’ psychological and behavioural responses. Previous research has shown that performance contexts involving direct observation and evaluation can significantly increase performer anxiety. To operationalize PA in empirical studies, researchers typically manipulate several audience-related variables.

First, audience size has been widely examined as a factor influencing arousal and performance. Studies have shown that larger audiences can intensify PSE and increase performance pressure (Baldwin and Clevenger, 1980; LeBlanc et al., 1997; Lemasson et al., 2018). Second, audience identity plays an important role in shaping performers’ expectations of evaluation. Different types of observers, such as teachers, peers, critics, or adjudicators, vary in perceived authority and evaluative significance (Cox, 1968; Leglar, 1978; Yoshie et al., 2009; Papageorgi et al., 2013; Lipton et al., 2020). Third, the performance setting may shape how performers interpret audience presence. Formal contexts such as examinations or competitions often heighten perceived evaluative pressure compared with informal rehearsal settings (Abel and Larkin, 1990; Konijn, 1991; Tartalone, 1992). Finally, perceived evaluative authority may further shape performers’ responses to physically present audiences. Observers perceived as highly knowledgeable or professionally authoritative may amplify evaluative apprehension and increase performance anxiety (Fancourt et al., 2015; Chanwimalueang et al., 2017; Aufegger and Wasley, 2018).

Together, these variables provide multiple methodological pathways through which PA can be systematically manipulated and assessed in experimental studies examining the relationship between audience presence and MPA.

#### 3.2.2 Inducing alternative attendance (AA)

AA is commonly operationalized through mediated conditions that imply the possibility of observation and future evaluation. Experimental studies frequently employ recording technologies to simulate evaluative listening contexts. For example, tape recorders have been widely used in experimental settings to create conditions of perceived evaluation (Fredrikson and Gunnarsson, 1992; Brotons, 1994). Similarly, video recording equipment can generate awareness that a performance will be reviewed at a later time, thereby inducing anticipatory evaluative pressure (Biasutti et al., 2016). Earlier studies have also demonstrated that the mere presence of recording devices can heighten performers’ sense of being observed and increase evaluation-related anxiety (Abrams and Manstead, 1981).

Researchers may also manipulate performers’ expectations regarding the identity or expertise of distant listeners. For example, Studer et al. (2012) increased perceived evaluative pressure by informing participants that their performances would be heard by “music adepts,” thereby enhancing perceived audience authority. In a related approach, Gomez et al. (2018) instructed participants to imagine that ordinary listeners were in fact musically knowledgeable connoisseurs, effectively amplifying the perceived evaluative stakes of the performance.

Recent technological developments have extended these approaches through immersive performance simulations. Virtual reality (VR) environments and simulated performance spaces enable researchers to recreate audience conditions without physically present observers. These technologies have been shown to elicit psychological and physiological responses comparable to those observed in live performance contexts (Williamon et al., 2014; Aufegger and Wasley, 2020), providing flexible methods for manipulating perceived audience presence while maintaining experimental control. These findings align with evidence suggesting that mediated or remote audiences can provoke measurable levels of MPA (Ryan et al., 2025). Even without physically present observers, performers may anticipate future judgment from distant listeners, thereby sustaining psychological pressure in mediated contexts.

Overall, these findings demonstrate that AA can be systematically induced through a range of mediated observation conditions. These methods provide practical tools for examining how technologically mediated audiences influence performers’ psychological responses and MPA.

#### 3.2.3 Inducing and assessing imaginative attendance (IA)

IA can be experimentally induced by asking performers to imagine an observing or evaluating audience, such as a teacher, examiner, peer group, or concert audience. Researchers may manipulate the audience’s evaluative orientation, authority, and consequences by presenting it as supportive, neutral, or critical. Control conditions should distinguish IA from general performance imagery, private practice, and no imagery.

Manipulation checks should assess audience vividness, psychological presence, identity, and perceived evaluation. IA may also be measured as a state during a specific performance or as a trait-like tendency across practice and performance. General imagery ability may be controlled statistically but should not be treated as IA itself.

### 3.3 Measurement of appraisal, anxiety, and performance responses

The impact of audience presence on performers’ responses to evaluative conditions can be assessed using multiple measurement approaches, allowing researchers to capture complementary perspectives on emotional, cognitive, and performance-related processes.

First, psychological responses can be assessed using self-report questionnaires. Common instruments include the MPA Inventory, which measures anxiety specific to musical performance contexts (Gomez et al., 2018). Measures of social anxiety and state anxiety may also be used to assess performers’ immediate emotional responses to audience-related situations (Gomez et al., 2018; Aufegger and Wasley, 2020). These questionnaires provide insight into performers’ subjective experiences of evaluative pressure during performance.

Second, physiological indicators can be used to assess performers’ autonomic and neuroendocrine responses to audience presence. Previous studies have examined physiological arousal through measures such as heart rate and heart rate variability, which reflect activation of the autonomic nervous system (Brotons, 1994; Chanwimalueang et al., 2017; Aufegger and Wasley, 2020). Other physiological markers include neuroendocrine responses associated with the hypothalamic–pituitary–adrenal (HPA) axis and the sympathetic–adrenal–medullary (SAM) system, both of which are involved in stress and anxiety responses (Fredrikson and Gunnarsson, 1992; Gomez et al., 2018; Aufegger and Wasley, 2018). Additional indicators, such as electromyographic activity and skin conductance, can provide further information about muscular tension and physiological arousal during performance (Yoshie et al., 2009).

Finally, behavioural and performance-based measures provide objective indicators of how anxiety influences observable aspects of performance (Barfield and Weghorst, 1993). Researchers have analysed performance accuracy and error frequency to assess the effects of audience pressure on musical execution (Abrams and Manstead, 1981). In addition, behavioural cues such as body movement patterns and eye contact may reflect performers’ levels of confidence, tension, and engagement during performance (Biasutti et al., 2016).

## 4 Discussion

This paper develops a multilevel framework for understanding audience-related processes in music performance anxiety (MPA) by distinguishing external audience conditions from internally generated audience representations. Physical Attendance (PA) and Alternative Attendance (AA) describe external configurations of audience access, whereas Imaginative Attendance (IA) refers to the internally generated or reactivated representation of specific or generalized others. These components are conceptually distinct and may operate independently or in combination, while also interacting dynamically across performance experiences.

The framework further proposes that audience-related anxiety is shaped not simply by the objective presence or absence of observers, but by how audience-related information is represented, interpreted, and appraised. Externally Assigned Identity (EAI) provides contextual information about who the audience is, while Perceived Evaluative Identity (PEI) reflects the evaluative meaning attributed to that audience by the performer. Perceived Social Evaluation (PSE) then integrates evaluative salience, evaluative stakes, and coping appraisal into an overall evaluative state. This structure helps explain why similar audience conditions may produce different psychological and performance outcomes across individuals and why evaluative pressure may persist in mediated or solitary contexts.

The framework also provides a basis for empirical testing by specifying both direct and indirect routes through which audience conditions may contribute to PSE. PA and AA may influence PSE directly through current audience-related cues and indirectly by activating or modifying IA. Existing IA may, in turn, shape the interpretation of subsequent physical or mediated audience conditions. Audience identity therefore informs how audience-related inputs are interpreted, whereas PSE integrates their evaluative relevance, consequences, and perceived manageability. These interacting processes offer a more flexible account of audience effects than models based exclusively on physical co-presence or linear mediation.

The following discussion considers the implications of the proposed framework in two respects. Section 4.1 examines its theoretical contribution to existing accounts of audience presence, evaluation, and MPA, whereas Section 4.2 outlines provisional practical implications for music education, performance preparation, and intervention.

### 4.1 Theoretical implications

#### 4.1.1 Audience presence as a multilevel representational system

The present framework reconceptualizes audience presence as a multilevel system comprising external audience conditions and internal audience representations. This conceptualization distinguishes audience presence from psychological appraisal: external conditions and internal representations function as audience-related inputs whose evaluative significance is constructed through audience identity and PSE.

IA functions as an internally generated or reactivated audience representation shaped by previous experiences, anticipated judgment, and socially learned expectations. Repeated encounters with evaluative roles may influence both the content of these representations and the PEI attributed to them. Performers whose internally represented audiences are perceived as supportive, or constructively evaluative may be more likely to regard performance demands as manageable. In contrast, representations associated with highly demanding evaluative identities may increase evaluative salience and stakes.

This distinction offers a possible explanation for individual variability in MPA. Performers exposed to similar external audience conditions may differ in the audiences they mentally foreground, the evaluative identities they attribute to those audiences, and their perceived capacity to cope with judgment. The psychological effects of audience presence may reflect the interaction between the external performance environment, the content of IA, the PEI assigned to represented audiences, and the resulting configuration of PSE.

#### 4.1.2 Extending established MPA and audience-based theories

The present framework extends established MPA models by specifying an audience-related representational and appraisal pathway within their broader etiological and temporal accounts. In relation to Barlow (2000) and Kenny (2011), it does not propose that audience presence independently causes MPA. Rather, PA, AA, and IA provide proximal inputs whose effects depend partly on performers’ pre-existing vulnerabilities, prior evaluative learning, perceived coping resources, and the identities attributed to actual or represented audiences. In relation to Papageorgi et al. (2007), the recursive interaction among PA, AA, and IA offers a more detailed account of how audience-related information may be anticipated before performance, updated during performance, and internalized following performance. Recent cross-sectional evidence provides a relevant but only partial point of comparison: Liu (2026) found that audience reaction was strongly associated with MPA, whereas the proposed mediation through performing conditions and moderation by performance-related mental-health beliefs were not supported. These findings underscore the relevance of audience-related information while leaving the mechanisms through which it is appraised unresolved. The present framework therefore elaborates the audience-specific mechanisms operating within broader developmental and temporal models of MPA.

Building on the social facilitation account introduced earlier, the present framework extends social facilitation theory by proposing that audience-related arousal is not inherently facilitative or inhibitory. PA, AA, and IA provide audience-related inputs whose effects depend partly on the configuration of evaluative salience, evaluative stakes, and coping appraisal within PSE. Metacognitive regulation subsequently influences whether performers remain dominated by threat-focused monitoring or redirect attention towards task execution and expressive goals (Eysenck et al., 2007; Jamieson et al., 2010). This account complements rather than replaces explanations based on task difficulty, familiarity, and dominant responses.

The framework also extends evaluation apprehension theory by proposing that IA can sustain anticipated judgment even when no audience is currently observing the performer. Internal representations of teachers, adjudicators, peers, critics, or anticipated listeners may remain psychologically active during solitary practice, preparation, and recording (Rapee and Heimberg, 1997). Evaluation apprehension may arise from socially derived audience representations that have been internalized or prospectively constructed, as well as from observers who are currently present or technologically connected.

The framework further contributes to social-presence accounts by distinguishing technological cues from the audiences they represent. Cameras, recording indicators, and platform interfaces may signal access by future or remote audiences, whereas livestream and virtual environments may create varying degrees of social presence (Lombard and Ditton, 1997; Williamon et al., 2014). The psychological effects of these cues depend partly on the EAI attached to the represented audience and the PEI constructed by the performer. By incorporating these identity-based processes, the framework links social presence to interpretation and appraisal across live, mediated, and solitary performance contexts.

### 4.2 Practical implications

The framework has potential implications for music education, performance preparation, and intervention. Because IA, PEI, and PSE have not yet been empirically or clinically validated, the following implications should be treated as theory-informed directions rather than established pedagogical or treatment recommendations.

#### 4.2.1 Early music training: teachers as formative audiences

Early Western classical instrumental training is an important developmental context in which musicians learn what it means to be heard, observed, and evaluated. Although MPA has been documented across child, adolescent, adult, and professional performers, young musicians vary considerably in how they interpret evaluative situations (Boucher and Ryan, 2011; Papageorgi, 2021). In this context, the instrumental teacher may be understood not as the student’s first literal audience, but as one of the earliest recurrent and socially significant evaluative figures in musical development. One-to-one lessons vary in interpersonal control, responsiveness, questioning, and instructional scaffolding (Creech, 2012), and autonomy-supportive teaching has been associated with better student well-being than controlling teaching behaviours (Bonneville-Roussy et al., 2020). Repeated performance experiences may also shape children’s stress responses, while aversive experiences accompanied by negative cognitions may increase later vulnerability to MPA (Boucher and Ryan, 2011; Osborne and Kenny, 2008). This does not mean that criticism or corrective feedback directly causes MPA. Rather, recurrent evaluative interactions may shape how students interpret mistakes, bodily arousal, personal competence, and the anticipated responses of future audiences.

Within the present framework, the teacher is a physically present audience-related source whose EAI is associated with expertise and evaluative authority. The psychological significance of this role depends on the student’s PEI: the same teacher may be experienced as a supportive guide, an unpredictable critic, or an authority whose approval is necessary for musical legitimacy. Through repeated interaction, this PEI may become internalized as an evaluator within IA and later reactivated during solitary practice, examinations, auditions, or public performance. PSE describes the proximal appraisal through which this representation becomes psychologically consequential.

This account offers more specific pedagogical implications than simply recommending that teachers be encouraging. Teachers may reduce the likelihood that feedback is interpreted as global personal judgment by separating technical errors from personal competence, normalizing mistakes as part of skill development, providing predictable and process-focused feedback, and introducing lower-stakes performance opportunities gradually. They may also help students reflect on whom they imagine listening during practice, what standards they attribute to this represented audience, and whether its PEI is supportive, neutral, or critical.

These strategies are consistent with preparation, positive framing, simulation, and anxiety-management practices identified in research on teachers’ approaches to MPA (Mazzarolo et al., 2023). Within this framework, their value lies in their potential to shape the PEI attributed to teachers and other evaluators represented within IA, thereby influencing the subsequent configuration of PSE. Longitudinal research is needed to test whether teaching climate and feedback style predict later MPA through changes in IA, PEI, and PSE.

#### 4.2.2 Potential implications for intervention

The framework intersects with two established approaches relevant to MPA: exposure-based interventions addressing avoidance of evaluative performance situations and cognitive approaches informed by models of threat interpretation, self-focused attention, and fear of negative evaluation (Appel, 1976; Wells et al., 1995; Rapee and Heimberg, 1997; Kaczkurkin and Foa, 2015). It adds an audience-specific level of analysis by suggesting that intervention may need to address not only external audience conditions, but also the audience representations, identities, and appraisal processes through which those conditions acquire evaluative meaning.

First, graduated exposure may help performers revise their responses to PA and AA. Exposure hierarchies could progress from individually calibrated lower-demand situations, such as self-recording or simulated audiences, to informal performances and more explicitly evaluative live settings. Repeated stage exposure has been associated with reductions in physiological arousal and performance errors (Appel, 1976; Candia et al., 2023). Within the present framework, however, the difficulty of an exposure condition may depend not only on audience size, physical proximity, or performance format, but also on evaluative salience, perceived stakes, PEI, and coping appraisal. A supportive peer, an instrumental teacher, and an adjudication panel may produce different appraisals within otherwise similar performance environments. Exposure hierarchies may be more informative when they incorporate performers’ perceived evaluative hierarchies alongside objective audience conditions.

Second, exposure to external performance situations alone may be insufficient when anxiety is partly sustained by a recurrent or readily activated critical IA. Performers may continue to anticipate judgment in comparatively neutral situations when earlier evaluative encounters have been encoded as critical audience representations. Observer-perspective imagery may further sustain such concerns even when current external evidence is limited or ambiguous (Rapee and Heimberg, 1997). Cognitive and imagery-based methods could examine which audiences are mentally foregrounded, what standards or evaluative stances are attributed to them, and how these representations influence coping appraisal.

Where appropriate within established clinical practice, imagery rescripting may provide one method of exploring recurring representations of criticism, humiliation, or conditional approval and developing more differentiated interpretations of evaluative experiences. The purpose would not be to deny technical errors or remove appropriate artistic standards, but to reduce the tendency to interpret specific performance difficulties as global evidence of incompetence or diminished self-worth. Whether such methods produce measurable changes in IA or PEI remains an empirical question.

Third, interventions may address the appraisal and regulatory processes represented by PSE and metacognitive regulation. Anxiety may redirect attention towards audience reactions, bodily sensations, and excessive error monitoring, thereby interfering with goal-directed attentional control (Eysenck et al., 2007). Performers may benefit from practising the redirection of attention towards musical structure, technical execution, expressive intention, and communication. They may also learn to interpret physiological activation as potentially manageable or functional rather than as unequivocal evidence of failure (Jamieson et al., 2010, 2013).

The construed identity of an audience may also be examined, including whether observers are represented primarily as hostile judges, legitimate evaluators, attentive listeners, or participants in musical communication. These strategies may alter the configuration and consequences of PSE rather than simply reducing its overall level. For example, evaluative salience and stakes may remain high while coping appraisal becomes more favourable and attentional regulation becomes more task-oriented.

### 4.3 Limitations, boundary conditions, and future research

Although the present framework offers a multilevel account of audience-related processes in MPA, several limitations and boundary conditions should be acknowledged. In the interest of theoretical parsimony, the model prioritizes audience identity as a major interpretive component and focuses on audience-related appraisal rather than attempting to incorporate every factor associated with performance anxiety. These limitations define the current scope of the framework and identify directions for construct validation, empirical testing, and theoretical extension.

#### 4.3.1 Construct validity and empirical testing

A primary limitation is that IA, PEI, and PSE are newly proposed or newly specified constructs whose psychometric properties and empirical distinctiveness have not yet been established. IA should be distinguished from general performance imagery, observer-perspective imagery, self-criticism, self-evaluation based solely on personal standards, and Elkind’s imaginary audience. The discriminant validity of PEI should likewise be examined in relation to related socioevaluative constructs.

Further clarification is required regarding the temporal status of these constructs. IA may contain relatively stable audience representations while varying in its situational activation. PEI may reflect both established expectations and moment-to-moment updating during reciprocal interaction. PSE is proposed primarily as a context-sensitive appraisal state, although repeated appraisal patterns may produce relatively stable individual tendencies. Experience-sampling, repeated-measures, and longitudinal designs may be particularly useful for distinguishing stable representations from situational changes.

The internal structure of PSE also requires empirical examination. Research should determine whether evaluative salience, evaluative stakes, and coping appraisal are empirically distinguishable dimensions and whether PSE is best represented as a higher-order construct, a formative configuration, or a profile of interacting appraisal dimensions. Scale development should combine qualitative item generation with exploratory and confirmatory factor analysis, while also incorporating interviews, experimental manipulation, experience sampling, and tests of convergent, discriminant, and predictive validity.

Finally, the causal relationships proposed by the framework remain untested. Experimental studies could manipulate PA, AA, and IA independently or in combination while examining their effects on PEI, PSE, anxiety, attention, physiological activation, and performance. Mediation analyses could test whether PEI transmits the effects of EAI to evaluative salience and stakes, while moderation analyses could examine whether metacognitive regulation changes the relationship between PSE and performance responses. Longitudinal research is also needed to determine whether repeated PA and AA experiences contribute to the formation or modification of IA over time.

#### 4.3.2 Alternative appraisal mechanisms and individual differences

Although audience identity is treated as a major interpretive component of PSE, it should not be regarded as the sole basis of evaluative appraisal. Performers’ interpretations of audience-related cues may also be shaped by broader inferential. From an attributional perspective, audience reactions may influence appraisal depending on how performers explain the source and meaning of evaluative outcomes (Weiner, 1985). These interpretations may shape both the PEI assigned to an audience and the performer’s appraisal of available coping resources.

The framework also does not provide a complete account of individual differences associated with MPA. Factors such as trait anxiety, perfectionistic concern, intolerance of uncertainty, shame proneness, personality characteristics, and previous humiliating or traumatic performance experiences may affect how audience-related inputs are interpreted, internalized, and regulated (Frost et al., 1990; Hewitt and Flett, 1991; Kenny, 2011; Carleton, 2016). Although the model acknowledges self-efficacy and metacognitive regulation, it does not specify their trait-level structure, developmental origins, or interaction with broader personality characteristics.

Future research should therefore examine whether these variables function as antecedents of IA and PEI, predictors of coping appraisal, moderators of PSE-related responses, or independent contributors to MPA. For example, performers with higher self-efficacy may be more likely to appraise high-stakes evaluation as manageable, whereas those with elevated trait anxiety or perfectionistic concern may assign greater salience and consequences to the same audience condition. Testing these possibilities would help clarify the explanatory boundaries of the framework.

#### 4.3.3 Generalizability across performance contexts

A further boundary condition concerns the framework’s primary focus on solo musical performance. The model prioritizes audience-related evaluation and assumes a relatively distinguishable performer–audience relationship. Many musical activities, however, occur in collaborative settings, including chamber music, orchestral performance, choirs, and other ensembles.

In these contexts, performers encounter multiple and overlapping sources of evaluation. Fellow musicians may simultaneously function as collaborators, comparison targets, support sources, and evaluative audiences. Performers must also coordinate timing, expression, and behaviour while monitoring the expectations and reactions of musicians and external listeners (Williamon and Davidson, 2002; Keller, 2014). As a result, the boundary between performer and audience may become less stable than in solo performance.

Future research should examine how PSE operates within collective performance environments, where performance regulation may involve interpersonal coordination and shared attentional control (Keller, 2014; Ginsborg and King, 2012). In particular, studies could investigate whether evaluative appraisal is socially coordinated or co-regulated among ensemble members, whether shared performance goals reduce evaluative threat, whether hierarchical ensemble structures increase evaluative stakes, and how IA or PEI develops when several socially significant evaluators are present simultaneously.

#### 4.3.4 Cultural and genre-specific boundary conditions

The present framework is grounded primarily in Western classical performance, where formal assessment, canonical repertoire, technical precision, and audience scrutiny frequently occupy important roles. Although this context provides a coherent setting for developing the model, its generalizability to other musical traditions and performance cultures remains uncertain.

Audiences in some jazz, popular, community, and non-Western performance traditions may occupy more participatory, improvisatory, or collaborative roles than those typically associated with formal classical concert settings. Under such conditions, audience presence may be interpreted less exclusively as evaluation and more as communication, interaction, collective participation, or shared artistic production. However, these distinctions should not be reduced to a simple opposition between evaluative Western classical performance and participatory non-Western traditions, as substantial variation exists within every genre and cultural context.

The boundary between performer and audience may not carry equivalent meanings across cultures. The same EAI may produce different PEIs depending on local social norms and musical values. Cross-cultural and cross-genre studies should therefore examine whether the three dimensions of PSE retain the same structure and whether EAI, PEI, PA, AA, and IA operate similarly across performance traditions.

Comparative research may reveal alternative relationships among audience conditions, internal representations, and evaluative appraisal. Such work would help establish the cultural boundaries of the present framework and determine whether its proposed mechanisms generalize across diverse musical ecologies or require context-specific modification.

## 5 Conclusion: the nature of performance

This paper proposes that audience presence in MPA should be understood as a multilevel process involving physical, mediated, and imagined audiences. The psychological effects of these forms of presence depend not only on objective audience conditions, but also on how performers construe audience identity, appraise evaluative salience and stakes, assess their coping capacity, and regulate their subsequent responses. Audience-related MPA is neither a direct consequence of audience presence nor a purely internal phenomenon. Within the present framework, it may arise through interactions among external conditions, internal audience representations, perceived social evaluation, and metacognitive regulation. By specifying these interactions, the framework offers a testable account of why similar performance situations may contribute to anxiety, inhibition, or facilitation. Future experimental and longitudinal research is needed to examine the validity of the proposed constructs and the causal and temporal relationships among them.

## Data Availability

The original contributions presented in the study are included in the article/supplementary material, further inquiries can be directed to the corresponding author.

## In memoriam

This work is dedicated to the memory of MoMo, a quiet companion whose presence made difficult days gentler.
